# Tobramycin and vestibulotoxicity: retrospective analysis of four cases

**DOI:** 10.1136/ejhpharm-2020-002588

**Published:** 2021-03-22

**Authors:** S E M Vonk, E J M Weersink, C J Majoor, E M Kemper, the Amsterdam Mucociliary Clearance Disease (AMCD) Research Group

**Affiliations:** 1 Hospital Pharmacy, Amsterdam UMC Location AMC, Amsterdam, The Netherlands; 2 Respiratory Medicine, Amsterdam UMC Location AMC, Amsterdam, The Netherlands

**Keywords:** cystic fibrosis, tobramycin, vestibulotoxicity

## Abstract

Over a course of 7 months, four patients developed vestibulotoxicity after treatment with intravenous tobramycin. Since vestibulotoxicity is a serious adverse effect which can be irreversible, an investigation was undertaken to determine if there was a cause for the toxicity and whether the quality of care had been inadequate. In this period, 26 patients with cystic fibrosis were treated with tobramycin according to valid guidelines, of which four experienced acute dizziness which disrupted their daily activities. Two patients experienced irreversible bilateral vestibular hypofunction and two unilateral loss of the right labyrinth, with decreasing dizziness over time. No apparent cause for the vestibulotoxicity was found in these four patients and the simultaneous occurrence was not due to a lack in quality of care. Symptoms of dizziness and balance disorders should be recognised by patients and caretakers at an early stage so additional diagnostics can be done to prevent further deterioration.

## Background

Cystic fibrosis (CF) is an autosomal recessive genetic disease caused by mutations in the Cystic Fibrosis Transmembrane conductance Regulator (CFTR) gene. This results in dysfunction of the CFTR protein, a chloride channel located in the surface of epithelial cells which regulates chloride and bicarbonate transport, fluid and pH in tissues.^1 2^ The lungs of patients with CF are prone to recurrent infectious disease exacerbations for which intravenous antibiotics are often needed. It is important that exacerbations are effectively treated, since an increase in exacerbation rate is associated with further lung damage and increased mortality.^1^

A common pathogen in patients with CF is *Pseudomonas aeruginosa*. The first-choice treatment for *Pseudomonas aeruginosa* is the third-generation cephalosporin ceftazidime in combination with the aminoglycoside tobramycin. Aminoglycosides have the well-known risk of causing adverse effects, the most common being nephro-, oto- and vestibulotoxicity.^3^ Despite these risks, aminoglycosides are still used on a regular basis and therapeutic drug monitoring is performed by the hospital pharmacy to prevent toxicity and ensure efficacy.

Vestibulotoxicity is described as a ‘common’ (≥1/100 to <1/10) adverse effect of tobramycin^4^; however, in our hospital none of the CF physicians and pharmacists had observed this adverse effect of tobramycin in the past 15 years. Over the course of 7 months four patients in our hospital developed vestibulotoxicity after treatment with intravenous tobramycin. Three of them had CF and one had primary ciliary dyskinesia. Since vestibulotoxicity is a serious adverse effect which can be irreversible, a multidisciplinary team was established to investigate if there was a cause for the toxicity and whether the quality of care had been inadequate.

## Case presentation

From October 2015 to April 2016 a total of 26 patients with CF in our hospital were treated with intravenous tobramycin for their pulmonary exacerbation according to valid guidelines. Four patients (15.4%) suffered from vestibulotoxicity, experiencing acute dizziness which disrupted their daily activities. Two patients experienced irreversible bilateral vestibular hypofunction and the other two unilateral loss of the right labyrinth, with decreasing dizziness over time. The basic characteristics of the four cases are shown in table 1.

**Table 1:** Basic characteristics, diagnosis and symptoms of the four cases

	Case 1	Case 2	Case 3	Case 4
CF or PCD	PCD	CF	CF	CF
CF mutation	Carrying one CF mutation dF508	dF508/R117H	dF508/Y1092X	dF508/dF508
Gender	F	F	F	F
Age (years)	35	57	35	44
Weight (kg)	117	50	50	58
eGFR (mL/min)	>60	>60	>60	>60
Mean tobramycin dose (mg/kg/24 hours)	4.9	11.8	10.4	8.8
Treatment duration (days)	27	21	15	21
Clinical/outpatient administration	Outpatient	Clinical	Outpatient	Both
Time to vestibulotoxicity (days after first treatment day)	21	25	13	30
Unilateral/bilateral vestibulotoxicity	Bilateral	Bilateral	Unilateral	Unilateral
Reversible/irreversible vestibulotoxicity	Irreversible	Irreversible	Reversible	Reversible
Diagnosis of vestibular hypofunction (electro-nystagmography (ENG))	Bilateral vestibulopathy	No central vestibular hypofunction, bilateral non-irritable labyrinths	No central vestibular hypofunction, non-irritable right labyrinth	No central vestibular hypofunction, less irritable right labyrinth
Description of symptoms	Dizziness, imbalance, reduced memory and concentration, increased imbalance with head movements	Dizziness, nausea, vomiting, increased dizziness with head movements, unable to walk, pressing headache, slurred speech	Dizziness, imbalance, increased dizziness with head movements, nausea and vomiting in the morning	Dizziness, cannot climb stairs, drive, cycle, walk alone
Follow-up of symptoms	Rehabilitation process, questioning symptoms, symptoms partly disappear and are stable, dependent on mobility scooter	Rehabilitation process, questioning symptoms, symptoms remain, dependent on mobility scooter	Questioning symptoms, symptoms partly disappear	Questioning symptoms, physiotherapy, disappear over time
Diagnosis hearing loss (audiogram)	Conductive hearing loss indicated by PCD	Bilateral high frequency perceptual loss	Unilateral left high frequency perceptual loss	Normal hearing

CF, cystic fibrosis; PCD, primary ciliary dyskinesia.

## Investigations

The Medical Ethical Committee of our hospital provided a formal letter of no objection to perform the study, as consent of patients is not required in the Netherlands for retrospective studies. The patients did not object to the use of their data. For the four cases, the indication for the use of tobramycin, prescriptions, doses and other possible causes (comedication, comorbidities) were evaluated. Peak and trough concentrations of tobramycin were checked to determine whether they were within the reference ranges of 25–35 mg/L and <0.5 mg/L, respectively. The validation reports of the apparatus on which tobramycin concentrations were measured were requested. Information regarding medication and supply chain were requested, as well as preparation protocols from the companies who prepare the intravenous medication for use at home. The manufacturer of tobramycin (Centrafarm, Etten-Leur) and Lareb (Netherlands Pharmacovigilance Centre) were asked if there had been more reports of this adverse reaction in 2015 and 2016. During the investigation, the hospital decided not to use tobramycin due to the severity of the adverse effect and to prevent occurrence of new cases.

## Treatment

In all the described cases, treatment with tobramycin was discontinued when the first symptoms of dizziness appeared. Subsequently, the ear, nose and throat (ENT) doctor was consulted to investigate the symptoms of vestibulotoxicity and hearing loss.

## Outcome and follow-up

Four patients developed vestibulotoxicity after treatment with intravenous tobramycin. Two patients had bilateral vestibulotoxicity, which was irreversible over time, and the other two patients had unilateral vestibulotoxicity, which was reversible over time. The diagnosis, symptoms and follow-up of symptoms are described in table 1.

For all four patients the indication for the use of tobramycin, prescriptions and doses were correct and other possible causes (comedication, comorbidity) could not be demonstrated. Therapeutic drug monitoring was performed according to our protocol, trough and peak concentrations were taken around the second tobramycin administration and in weeks 2 and 3 by the hospital pharmacy on validated equipment. Peak and trough serum concentrations of tobramycin were within the reference values of 25–35 mg/L and <0.5 mg/L, respectively. There were no deviations in the medication and supply chain. Preparation protocols were requested from the company who prepared the intravenous medication for use at home and no deviations were found. There were no reports of this adverse effect known by the manufacturer of tobramycin and only one report (2011) was known by Lareb.

## Discussion

There was no apparent cause of the vestibulotoxicity in these four patients and the simultaneous occurrence in our hospital was not due to a lack in the quality of care.

Only one published study has examined the occurrence of vestibulotoxicity with tobramycin in patients with CF. Scheenstra *et al* 5 found a high prevalence (30.4%) of vestibulotoxicity in patients with CF. In this study, electronystagmography (ENG) with caloric irrigation was performed in 23 patients with CF who were treated with intravenous tobramycin. ENG is often regarded as the gold standard test to diagnose vestibular hypofunction. Abnormal ENG results were found in seven patients (30.4%). However, only three patients reported symptoms of dizziness that matched vestibular loss. This finding suggests that abnormal ENG results can be found, but patients do not (yet) experience symptoms of vestibulotoxicity. This remarkable difference can possibly be explained by the vague presenting symptoms of vestibulotoxicity and its slow onset, as well as the limitations of the caloric test itself which can only examine the function of the horizontal semicircular canals and superior parts of the vestibular nerves.^6^ Symptoms of vestibulotoxicity such as imbalance, instability, headache and dizziness may not always be noticed, are linked to another underlying disease or are discovered at a late stage because patients are often less mobile during treatment.^5 7^ Therefore, vestibulotoxicity is often misdiagnosed. Additionally, a wide variety of different diagnostic tests are used, but there is no standard protocol which describes the screening for vestibulotoxicity during tobramycin treatment.^7^ Symptoms of imbalance like dizziness should be recognised by patients and caretakers (eg, physicians and nurses) at an early stage so that additional diagnostics can be done to prevent further deterioration.

For these four cases, the doses of tobramycin were correct, therapeutic drug monitoring was performed and peak and trough concentrations were within the reference ranges. Thus, no clear dose-related toxicity could be found. It was not possible to calculate the total cumulative tobramycin exposure for these patients as they had been treated with tobramycin for a long period of time, even before the electronic patient record was implemented. Scheenstra *et al* were also unable to find a clear association between the total cumulative exposure to tobramycin treatment and vestibulotoxicity.^5^ Further research is needed to define the possible characteristics that underlie this toxicity. Pauna *et al* 8 found histopathological changes due to exposure to aminoglycosides in the vestibular structures of human temporal bone specimens from deceased donors with CF. However, one can speculate that the vestibular structures are intrinsically changed in patients with CF. To prove this, case–control studies are needed to exclude this intrinsic CF phenomenon.

In our hospital, the treatment of patients who did not experience symptoms with tobramycin was restarted with extra alertness by caretakers and patients for these symptoms, and no vestibulotoxicity has since been reported.

**Learning points**

- Even though therapeutic drug monitoring was performed with adequate peak and trough concentrations, four patients developed vestibulotoxicity after treatment with intravenous tobramycin over a short time period.
- There seems to be no clear association between the total cumulative exposure to tobramycin treatment and the occurrence of vestibulotoxicity.
- Patients and caretakers should be aware of early symptoms of vestibulotoxicity to prevent deterioration.

## Data Availability

Data are available upon request.
